# Sexual dimorphisms in innate immune activation markers in predementia Alzheimer’s disease

**DOI:** 10.1093/braincomms/fcaf161

**Published:** 2025-04-25

**Authors:** Stephanie Knudtzon, Kaja Nordengen, Lene Pålhaugen, Berglind Gísladóttir, Jonas Jarholm, Geir Bråthen, Ragnhild Eide Skogseth, Knut Waterloo, Per Selnes, Tormod Fladby, Bjørn-Eivind Kirsebom

**Affiliations:** Department of Neurology, University Hospital of North Norway, 9038 Tromsø, Norway; Department of Psychology, Faculty of Health Sciences, UiT The Arctic University of Norway, 9037 Tromsø, Norway; Department of Neurology, Akershus University Hospital, 1478 Lørenskog, Norway; Institute of Clinical Medicine, University of Oslo, 0318 Oslo, Norway; Department of Neurology, Akershus University Hospital, 1478 Lørenskog, Norway; Institute of Clinical Medicine, University of Oslo, 0318 Oslo, Norway; Department of Neurology, Akershus University Hospital, 1478 Lørenskog, Norway; Clinical Molecular Biology (EpiGen), Medical Division, Akershus University Hospital and University of Oslo, 1478 Lørenskog, Norway; Department of Neurology, Akershus University Hospital, 1478 Lørenskog, Norway; Institute of Clinical Medicine, University of Oslo, 0318 Oslo, Norway; Department of Neuromedicine and Movement Science, Faculty of Medicine and Health Science, Norwegian University of Science and Technology, 7491 Trondheim, Norway; Department of Neurology and Clinical Neurophysiology, Trondheim University Hospital, 7491 Trondheim, Norway; Department of Geriatric Medicine and the Neuro-SysMed Centre, Haraldsplass Deaconess Hospital, 5021 Bergen, Norway; Department of Clinical Science, University of Bergen, 5021 Bergen, Norway; Department of Neurology, University Hospital of North Norway, 9038 Tromsø, Norway; Department of Psychology, Faculty of Health Sciences, UiT The Arctic University of Norway, 9037 Tromsø, Norway; Department of Neurology, Akershus University Hospital, 1478 Lørenskog, Norway; Department of Neurology, Akershus University Hospital, 1478 Lørenskog, Norway; Institute of Clinical Medicine, University of Oslo, 0318 Oslo, Norway; Department of Neurology, University Hospital of North Norway, 9038 Tromsø, Norway; Department of Psychology, Faculty of Health Sciences, UiT The Arctic University of Norway, 9037 Tromsø, Norway; Department of Neurology, Akershus University Hospital, 1478 Lørenskog, Norway; Institute of Clinical Medicine, University of Oslo, 0318 Oslo, Norway

**Keywords:** sex differences, inflammation, innate immune system, cerebrospinal fluid

## Abstract

Females have an increased risk of developing Alzheimer’s disease (AD). The innate immune system plays a key role in AD pathology, and sex differences in innate immune responses may contribute to differences in disease risk and progression. This study investigated sex differences in innate immune responses among participants without cerebrospinal fluid (CSF) determined amyloid pathology [A–; cognitively normal (CN), *n* = 83] and those with amyloid pathology (A+, *n* = 202), further stratified into preclinical (CN with A+, *n* = 72) and mild cognitive impairment (MCI with A+, *n* = 130). Participants were drawn from the Norwegian Dementia Disease Initiation cohort (*n* = 285). We measured plasma glial fibrillary acidic protein (GFAP) and CSF concentrations of nine innate immune markers: soluble triggering receptor expressed on myeloid cells 2 (sTREM2), monocyte chemoattractant protein 1 (MCP-1), fractalkine, chitinase 3-like 1 (YKL-40), clusterin, interferon gamma (IFN-γ), interleukin-6 (IL-6), IL-10, and IL-18. Linear regression was used, adjusted for multiple comparisons using the false discovery rate. In A+ cases (*n* = 202, females = 105), females had lower MCP-1 (*P*  *<* 0.01), IL-6 and IL-18 (both *P*  *<* 0.05) than males, while no sex differences were observed in A– cases (*n* = 83, females = 39). Among A+ participants, no sex differences were observed in CN cases (*n* = 72, females = 37), but females (*n* = 68) with MCI had lower MCP-1 and IL-6 (both *P*  *<* 0.05) than males (*n* = 62) with MCI. Moreover, A+ females exhibited stronger positive associations between sTREM2 and clusterin with CSF total tau (*P* < 0.001; *P* < 0.05) and Neurofilament light chain (*P* < 0.01; *P* < 0.01) than males. These findings suggest sex-specific differences in innate immune responses, which may contribute to disease progression in amyloid-positive individuals.

## Teaser

Knudtzon *et al*. have studied sex differences in the innate immune system's role in Alzheimer's Disease (AD). Their findings point to sex potential differences in cerebrospinal fluid innate immune responses that may contribute to the understanding of the different patterns of disease progression in females and males with AD.

## Graphical Abstract

**Graphical Abstract:**
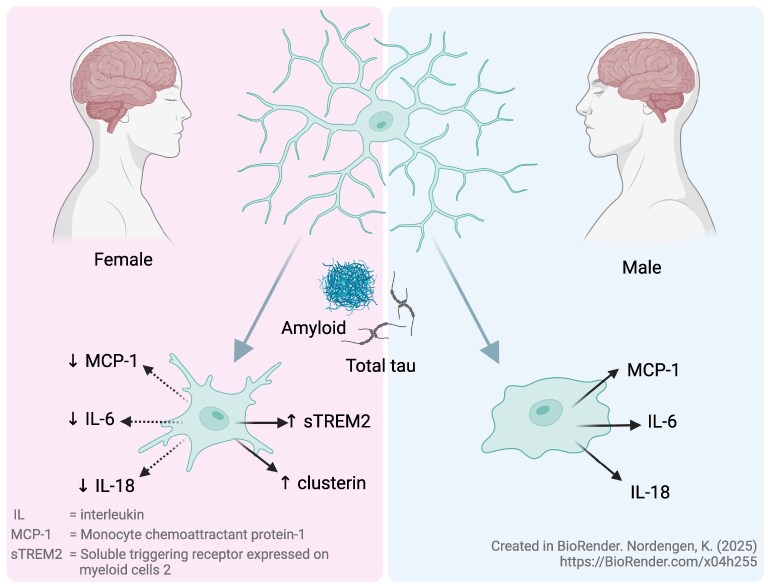

## Introduction

In Alzheimer's Disease (AD), neuropathological changes characterized by the accumulation of amyloid beta (Aβ) plaques and neurofibrillary tangles start several years before clinical symptoms,^1^ and may be detected by decreased cerebrospinal fluid (CSF) Aβ_1–42_ to Aβ_1–40_ (Aβ_42/40_) ratios and elevated concentrations of phosphorylated tau (p-tau).

Glial cells play key roles in brain immune activation.^2^ In AD, microglia and astroglia are observed around Aβ plaques and may have a role in protein clearance.^3^ This aligns with the hypothesis of an early protective role, where microglial activation may clear Aβ species and reduce amyloid load,^4,5^ followed by a later detrimental role of innate immune activation contributing to inflammation.^5-8^ We previously identified glial hypoactivation in Aβ-positive (A+) cases and increased glial activation corresponding with higher CSF total tau (t-tau) and p-tau concentrations.^9^ However, the transition from a protective to a potentially neurotoxic role of microglia is not well understood and the mechanisms are still unclear.^10^

Sex differences in AD have been described, including differences in incidence and prevalence,^11,12^ with females comprising approximately two-thirds of individuals with clinical AD.^11^ Moreover, females have more frequent amnestic mild cognitive impairment (MCI),^13^ experience a more rapid decline in cognition and function,^14^ and progress from MCI to AD dementia at a faster rate.^15-17^ Furthermore, sex differences likely also encompass immune regulations more broadly, as suggested by the much higher number of females with autoimmune diseases.^18-20^ Additionally, studies have demonstrated sex differences in gene expression and regulation of microglia.^21^ A study on peripheral immune response found that females with AD had lower average levels of innate immune markers (TNF-α, IL-10 and IL-1β), a weaker leukocyte response and reduced cytokine production in response to viral infection compared to males with AD. Importantly, no sex differences were observed in the healthy control group.^22^ In spite of this, the potential role of the innate immune system in AD sexual dimorphism has only been studied to a limited extent.

For these reasons, evaluating possible sex differences in innate immune responses is particularly relevant in AD research and may have important implications for therapy, including anti-amyloid therapies. Trial data suggest a differential response, with females possibly experiencing less benefit, and a potential role of sex-linked differences in innate immune reactions cannot be ruled out.^23^

We and others have found that sex is a significant covariate for several CSF innate immune activation markers across the AD continuum.^9,22,24^ In this study, we aim to determine whether sex differences are present in cognitively normal (CN) males and females without amyloid pathology, or if they emerge during the preclinical (CN) and prodromal (MCI) phases of AD (A+). To explore this, we analyze the expression patterns of nine CSF innate immune markers: soluble triggering receptor expressed on myeloid cells 2 (sTREM2), monocyte chemoattractant protein 1 (MCP-1), fractalkine, chitinase 3-like 1 (YKL-40), clusterin, interferon gamma (IFN-γ), interleukin-6 (IL-6), IL-10 and IL-18, along with one plasma innate immune marker, glial fibrillary acidic protein (GFAP). Building on our previous research showing increased glial activation linked to tau pathology (both p-tau and t-tau),^9^ we further assess whether CSF neurodegeneration markers t-tau and neurofilament light chain (NfL) associate differently with innate immune markers in males and females.

## Materials and methods

### Study design

For the purposes of our study, 285 participants from the Norwegian multicenter study Dementia Disease Initiation (DDI) were included and classified according to the presence or absence of pathological Aβ_42/40_ ratios, with 83 Aβ negative (A−), and 202 A+. In order to ensure that all females were postmenopausal and thereby ruling out any possible beneficial effects of oestrogen,^25^ we here included participants older than 60 years (mean menopause age in Norway is 52 years)^26^ and excluded participants on hormone replacement therapy (*n* = 18). Apart from these adaptations, we followed the standard DDI exclusion criteria: history of brain trauma or brain disorders, severe psychiatric disease, severe somatic disease that might influence cognitive functions or intellectual disability and other developmental disorders. Participants were recruited between 2013 and 2023 from memory clinics at university hospitals across Norway and through advertisements in media (newspapers or news bulletins). Healthy controls were recruited from spouses of patients with symptoms of cognitive disorders, advertisements in media, and from patients with self-reported normal cognition who completed lumbar puncture in connection with orthopaedic surgery. The lumbar punctures collected in relation to surgery were performed before anaesthesia was administered, and before surgery began. In the present study, participants were either CN or diagnosed with MCI (*n* = 130). The CN group included participants recruited as controls (RaC, *n* = 52) and those reporting subjective cognitive decline (SCD, *n* = 103). We have previously demonstrated that there are no significant differences in linear cognitive performance over time between RaC and SCD within the DDI cohort,^27^ and no statistical differences in age, CSF t-tau or NfL concentrations, baseline cognitive performance, sex distribution or apolipoprotein E epsilon 4 (*APOE-ɛ4)* status between RaC and SCD.^28^ SCD was defined according to the SCD-I framework, as persons performing normally on objective neuropsychological tests, but subjectively experiencing cognitive difficulties within a cognitive domain.^29^ NIA-AA criteria were used to define MCI,^30^ where the MCI group had concerns regarding their cognitive function, either self-reported or reported by spouses or clinicians, and scored lower than expected in one or more cognitive domains but were independent in daily functional ability and did not fulfil the criteria for dementia. Scores 1.5 standard deviation (*SD*) below the normative mean on either the Consortium to Establish a Registry for Alzheimer’s Disease (CERAD) Word List Task delayed memory subtest,^31^ Visual Object and Space Perception (VOSP) silhouettes,^32^ Trail Making Test part B (TMT-B)^33^ or Controlled Oral Word Association Test (COWAT)^34^ were used to define abnormal cognition. See Fladby *et al.*^35^ for further information regarding clinical assessment and procedures.

Nine participants in the A+ group were included as controls but exhibited below average performance on cognitive tests and were therefore reclassified as MCI. Although data collection was conducted across six locations in Norway (Akershus University Hospital; St. Olavs, University Hospital; Stavanger University Hospital; Haugesund Hospital; Betanien hospital; and University Hospital of North Norway), all participants underwent a standardized protocol including evaluation of patient medical history, blood and CSF collection, clinical and neurological examination, and neuropsychological testing. Notably, CSF sTREM2 measurements were available for all participants, while IFN-γ was analyzed only in a subsample as part of our previous study.^9^

### CSF and plasma markers

CSF was collected before noon in polypropylene tubes (Thermo Fisher Scientific, MA, USA) and centrifuged within 4 h at 2000g for 10 min at room temperature according to the BIOMARKAPD protocol.^36^ CSF and blood samples from each contributing DDI site were frozen and shipped to Akershus University Hospital for biobank storage and analysis. CSF t-tau was determined using the Innotest hTau Ag kit (Fujirebio, Ghent, Belgium) and analyzed at the Department of Interdisciplinary Laboratory Medicine and Medical Biochemistry. CSF NfL, Aβ_1–40,_ Aβ_1–42,_ Clusterin, MCP-1, IFN-γ, sTREM2, YKL-40, fractalkine, IL-6, IL-10, and IL-18 were measured using the QuickPlex SQ 120 system from Meso Scale Discovery (MSD, MD, USA) at the Department of Clinical Molecular Biology (EpiGen), as previously described.^9^ The sandwich ELISA method was used to analyze sTREM2, the method has been previously described by Suárez-Calvet *et al*.^37^ In all MSD analyses, the samples were analyzed in duplicate and reanalyzed if relative deviations (RDs) exceeded 20%. Quality control samples with an RD threshold of 15% were used to control for interplate and interday variation. Due to differences in the 9-plex and 4-plex setups for CSF IL-6, IL-18, MCP-1 and fractalkine, potential differences were assessed in statistical models. CSF NfL was measured in an R-plex format using the Human Neurofilament L Assay (K1517XR-2), while Aβ_1–40_ and Aβ_1–42_ in a multiplex setup using V-plex Aβ Peptide Panel 1 (6E10) kit (K15200E-1). The samples were pre-diluted 1:2 for both assays. The ratio of CSF Aβ_1–42_ to Aβ_1–40_ (Aβ_42/40_ ratio) was used to determine the presence or absence of Aβ plaque pathology. The cut-off (≤0.077) for Aβ_42/40_ ratio was determined following receiver operating characteristic (ROC) analysis with visual reads of [18F]-Flutemetamol PET scans as the standard of truth.^38^ Plasma GFAP was measured on the Simoa HD-X platform using the GFAP Discovery kit (Batch 10735) as per the manufacturer’s instructions (Quanterix, Billerica, MA). According to the kit insert, the lower limit of detection was 0.211 pg/mL, and the lower limit of quantification was 0.686 pg/mL. Samples were run in singlicate with an 8-fold dilution, and results were adjusted accordingly. Quality control samples were analyzed in duplicate at the start and end of each plate to assess precision. These measurements were performed at the Clinical Neurochemistry Laboratory, Sahlgrenska University Hospital, Mölndal, Sweden. Apolipoprotein E (*APOE*) genotyping was performed on EDTA blood samples, as previously described.^39^

### Statistical analysis

Statistical analyses were conducted using R v4.1.2,^40^ and figures were generated using the ‘*ggplot2’* and ‘*ggeffects’* packages.^41,42^ Independent-samples t-tests were used to assess age differences between our A– and A+ groups, whereas chi-square tests were used to assess differences in distributions of sex and *APOE-ɛ4* genotypes. First, we performed multiple linear regression with plasma and CSF immune markers as dependent variables and the sex × Aβ status interaction as an independent predictor. The ‘*emmeans*’ R package was used for pairwise between-group comparisons for each marker (*n* = 10 models). We extracted the comparisons of interest (sex differences within A+ and A− groups, *n* = 2 comparisons per model) and applied false discovery rate (FDR) adjustments (*n* = 20 comparisons). Second, a subanalysis within the A+ group was conducted. Here, the sex × cognitive status interaction was assessed, followed by similar pairwise comparisons for each marker. We extracted comparisons of interest (sex differences within A + CN and MCI groups, *n* = 2 comparisons per model) for each immune marker (*n* = 10 models, *n* = 20 comparisons) and applied FDR adjustments. Due to slight deviations from normality in regression residual diagnostics, CSF neurodegeneration markers were log-transformed prior to analysis. For all models, age, *APOE-ɛ4* carriership, CSF t-tau, and NfL were included as covariates, and the significance threshold was set at *P* < 0.05 following FDR adjustments. Finally, sex differences in the associations between innate immune markers and CSF neurodegeneration markers (t-tau and NfL) were analyzed within A+ cases using multiple linear regression models. An interaction term between sex and innate immune markers was included, with log-transformed CSF neurodegenerative markers as dependent variables. Age and *APOE-ɛ4* carriership were included as covariates. We also computed slopes for males and females separately using the ‘*emmeans*’ R package. The significance threshold was set at *P* < 0.05, and no adjustments for multiple comparisons were applied to these models.

For ease of comparison between models, standardized beta coefficients (β) are reported for all regression analyses. In addition, to assess potential differences between the 4-plex and 9-plex setups for IL-6, IL-18, MCP-1, and fractalkine (see CSF marker section above), we evaluated a random intercept in linear mixed models to account for setup differences for these markers. Since results and model fit were similar, we opted to proceed without these adjustments.^9^

### Ethics

The study was approved by the Regional Committees for Medical and Health Research Ethics in Norway and conducted in accordance with the guidelines provided by the Helsinki Declaration of 1964, revised in 2013, and the Norwegian Health and Research Act. All participants provided written informed consent before participating in the study.

## Data availability

Data from the DDI cohort are stored at Services for Sensitive Data (TSD) at the University of Oslo (UiO) and are not publicly available. However, anonymized data used in this study may be made available from the corresponding author upon reasonable request. No new software or code was generated for this study.

## Results

### Demographics

The A+ subjects were slightly older (3.5 years on average, *t* = 5.15, *P*  *<* 0.001) than the A– group and a higher frequency of *APOE-ɛ4* genotypes (72% versus 26.7%, *χ^2^* = 41.195, *P*  *<* 0.001). There were no significant differences in sex distributions between the groups (*χ^2^* = 0.327, *P*  *=* 0.568; see table 1 for details), and no sex differences in the frequencies of *APOE*-ɛ4 genotypes between A– males and A– females (*χ^2^* = 0.413, *P*  *=*  *0.521*), or between A+ males and A+ females (*χ^2^* = 0, *P*  *=*  *1*).

**Table 1:** Demographic, between-group comparisons of age, sex and APOE-ɛ4 carrier status

	Total (*n* = 285)						
	A– (83)			A+ (202)			A– versus A+ *t*/*χ^2^*/*(P)*
	Male (44)	Female (39)	Total	Male (97)	Female (105)	Total	
**Age** Mean (SD)	67.4 (5.35)	66.0 (5.22)	66.7 (5.31) [60–80]	70.0 (5.63)	70.8 (5.07)	70.4 (5.35) [60–83]	*t* = 5.15 (**<0.001**)
**Sex** *n* (%)	44 (50.7)	39 (49.3)	-	97 (49.1)	105 (57.1)	-	*χ^2^* = 0.327, (=0.568)
***APOE-ɛ4*** *n* (%)	14 (31.8)	9 (23.1)	20 (26.7)	68 (70.1)	73 (69.5)	126 (72)	*χ^2^* = 41.195, (**<0.001**)
**RaC** *n* (%)	15 (34.9)	16 (41)	31 (37.3)	7 (7.2)	14 (13.3)	21 (10.4)	^C^
**SCD** *n* (%)	29 (65.9)	23 (58.9)	52 (62.7)	28 (28.9)	23 (21.9)	51 (25.2)	^C^
**MCI** *n* (%)	0 (−)	0 (−)	0 (−)	62 (63.9)	68 (64.8)	130 (64.4)	^C^
**GFAP** mean (SD) [*n*]	127 (58) [38]	146 (64.9) [36]	136 (61.8) [74]	209 (130) [83]	231 (103) [90]	220 (117) [173]	^C^
**sTREM2** mean (SD) [*n*]	4.11 (1.18) [44]	3.83 (1.24) [39]	3.98 (1.21) [83]	4.92 (1.91) [97]	4.92 (1.64) [105]	4.92 (1.77) [202]	^C^
**MCP-1** mean (SD) [*n*]	515 (113) [38]	449 (88) [37]	482 (106) [75]	566 (141) [86]	492 (131) [88]	528 (141) [174]	^C^
**Fractalkine** mean (SD) [*n*]	1960 (560) [37]	1924 (560) [37]	1942 (557) [74]	2187 (521) [72]	2190 (683) [77]	2189 (608) [149]	^C^
**YKL-40** mean (SD) [*n*]	176 (52.6) [38]	160 (58.3) [38]	168 (55.7) [76]	211 (69.2) [87]	196 (70.7) [90]	203 (70.2) [177]	^C^
**Clusteirn** mean (SD) [*n*]	2091 (469) [38]	1799 (620) [39]	1943 (567) [77]	2471 (822) [78]	2280 (707) [89]	2369 (767) [167]	^C^
**IFN-γ** mean (SD) [*n*]	43.0 (28.9) [27]	62.6 (52.0) [20]	51.4 (41) [47]	46.4 (34.6) [46]	46.2 (35.0) [49]	46.3 (34.6) [95]	^C^
**IL-6** mean (SD) [*n*]	1.74 (0.68) [38]	1.61 (0.67) [37]	1.68 (0.68) [75]	1.79 (1.26) [86]	1.46 (0.62) [89]	1.62 (0.99) [175]	^C^
**IL-10** mean (SD) [*n*]	71.6 (33.6) [27]	87.9 (50.3) [20]	78.6 (41.8) [47]	83.7 (53.6) [46]	76.5 (39.8) [50]	79.9 (46.8) [96]	^C^
**IL-18** mean (SD) [*n*]	6.99 (2.74) [37]	6.12 (2.13) [37]	6.55 (2.48) [74]	7.13 (2.48) [86]	6.00 (2.42) [88]	6.56 (2.51) [174]	^C^
**T-tau** mean (SD) [*n*]	268 (63.1) [44]	247 (67.4) [39]	258 (65.5) [83]	607 (302) [97]	545 (275) [105]	575 (289) [202]	^C^
**NfL** mean (SD) [*n*]	3247 (1005) [39]	2596 (1689) [36]	2935 (1405) [75]	4873 (2180) [89]	3936 (2187) [93]	4394 (2228) [182]	^C^

Abbreviations: A+/−, positive or negative CSF marker for amyloid plaques; *APOE-ɛ4,* apolipoprotein E epsilon 4; GFAP, glial fibrillary acidic protein; IL-6, interleukin-6; MCI, mild cognitive impairment; MCP-1, monocyte chemoattractant protein 1; *n*, number of cases; NfL, neurofilament light chain; RaC, recruited as controls; SCD, subjective cognitive decline; SD, standard deviation; sTREM2, soluble triggering receptor expressed on myeloid cells 2; t, *t*-test statistics; t-tau, total tau; %, percentage; *χ^2^,* chi-square statistic; YKL-40, chitinase 3-like 1; c indicates no *post hoc* comparisons performed. Cognitive status and sex distribution for each CSF immune markers and neurodegeneration markers in the A– and A+ group.

As anticipated, the A + MCI group exhibited significantly poorer performance across all neuropsychological assessments compared to the A – CN reference group, as detailed in Supplementary Table 1. Notably, the most substantial deficits were observed in the CERAD word list delayed memory task (mean difference = 18.43, *P* < 0.001) and the Trail Making Test Part B (TMT-B) (mean difference = 16.51, *P* < 0.001).

### Potential sex differences in CSF MCP-1, IL-6 and IL-18 concentrations within the A+ group

**Figure 1:**
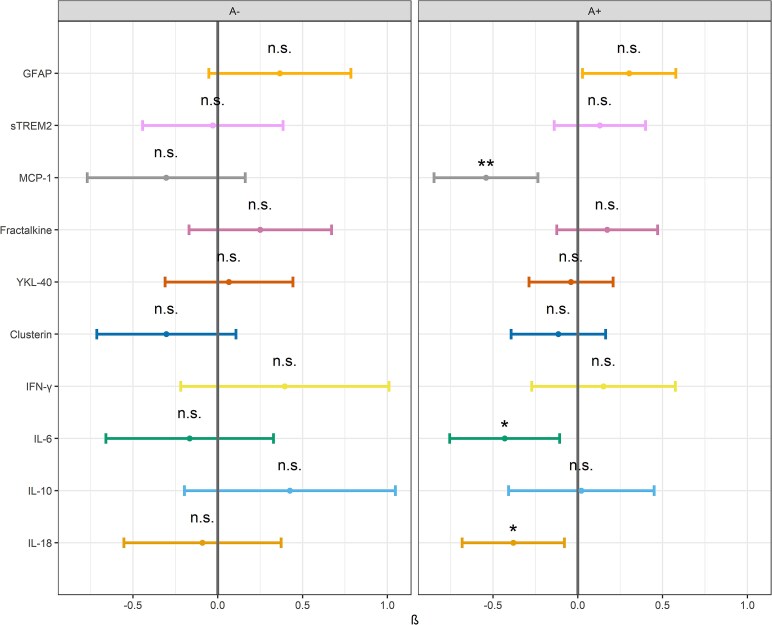
**Interaction effects of amyloid status and sex on innate immune markers:** Log-transformed and standardized (Z-log) concentrations of the 10 innate immune markers: plasma GFAP, CSF sTREM2, MCP-1, fractalkine, YKL-40, clusterin, IFN-γ, IL-6, IL-10 and IL-18 in the amyloid negative (A−, left) and amyloid-positive (A+, right) group. Horizontal bars show the 95% confidence intervals for each marker. Males are the reference (grey vertical bar). The statistical analyses conducted were multiple linear regression with plasma and CSF innate immune markers as dependent variables and the sex × Aβ status interaction as independent predictor. Covariates included were age, *APOE-ɛ4,* CSF t-tau and NfL. The analyses were conducted using the entire cohort (*n* = 285). * Indicates significance threshold of *P* < 0.05 and ** indicates significance threshold of *P* < 0.01.

After adjusting for covariates (age, *APOE-ɛ4* carriership, CSF t-tau and NfL), none of the models showed significant interaction effects between amyloid status and sex for our innate immune markers. Nevertheless, we opted to pursue FDR-adjusted *post hoc* analyses of sex differences within the A+ group. These analyses were suggestive of lower CSF MCP-1, IL-6 and IL-18 concentrations (*P*  *<* 0*.001*, *P*  *<* 0.05, and *P*  *<* 0.05 respectively) compared to males. In contrast, no significant sex differences were observed in the A– group. See Fig. 1 and Supplementary Table 2.

### Potential sex differences in CSF MCP-1 and IL-6 in individuals with MCI in the A+ group

**Figure 2:**
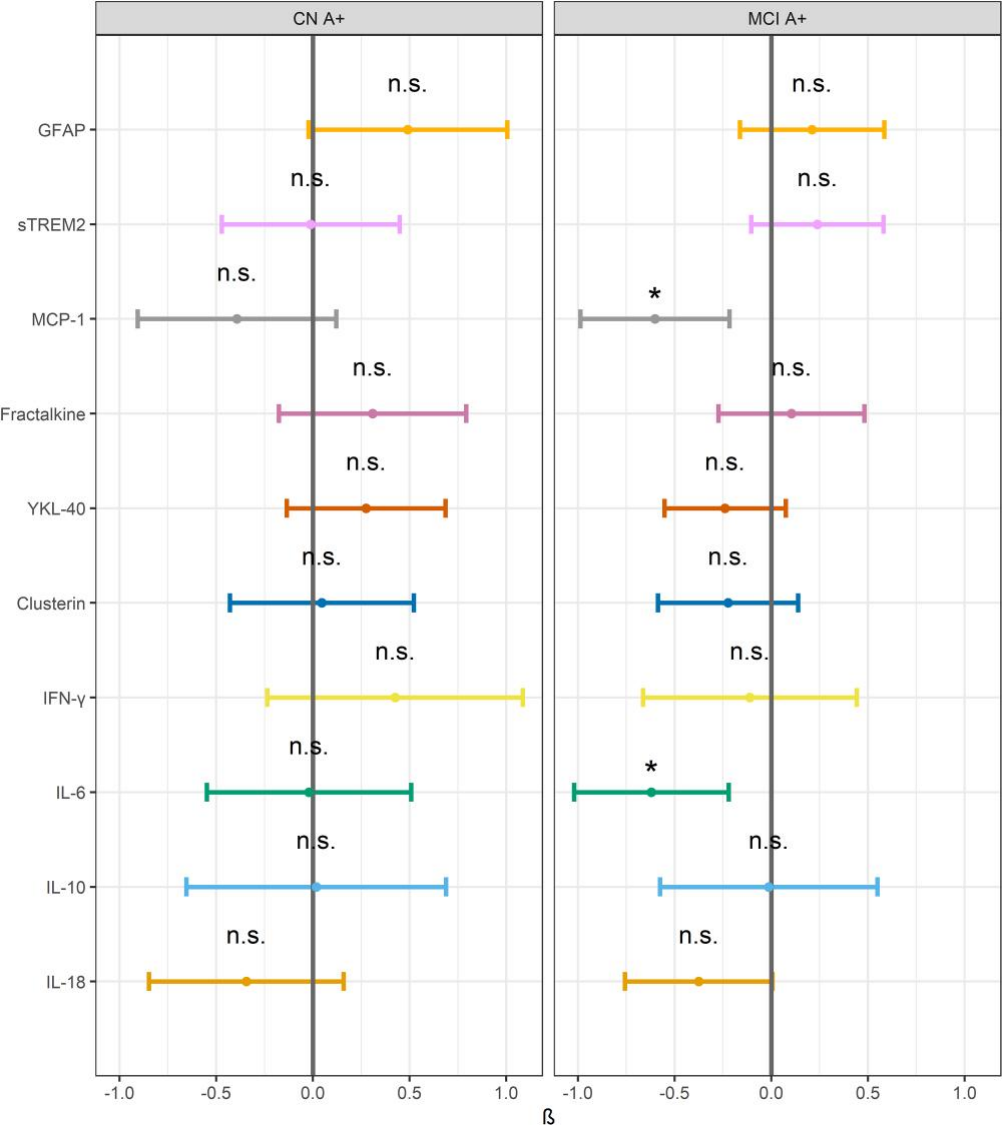
**Interaction effects of sex and cognitive status on innate immune markers in amyloid-positive individuals:** Log-transformed and standardized (Z-log) cerebrospinal fluid (CSF) concentrations of the ten innate immune markers: plasma GFAP, CSF sTREM2, MCP-1, fractalkine, YKL-40, clusterin, IFN-γ, IL-6, IL-10 and IL-18 in amyloid-positive cognitive normal (A + CN, left) and amyloid-positive mild cognitive impaired (A + MCI, right) group. Horizontal bars show the 95% confidence intervals for each marker. Males are the reference (grey vertical bar). The statistical analyses conducted were multiple linear regression within the A+ group (*n* = 202) with plasma and CSF innate immune markers as dependent variables and the sex × cognitive status interaction as independent predictor. Covariates included were age, *APOE-ɛ4,* CSF t-tau and NfL. * Indicates significance threshold of *P* < 0.05.

In our subanalysis of A + CN and MCI participants, only YKL-40 showed a significant interaction effect between cognitive status and sex. Nonetheless, FDR-adjusted *post hoc* analyses of sex differences within A + MCI participants suggested that females had lower CSF MCP-1 and IL-6 concentrations compared to males (both *P* < 0.05). However, no significant sex differences were observed in A + CN cases, see Fig. 2 and Supplementary Table 3. We also assessed sex-differential effects by including an interaction term between sex and cognitive status (sex × MCI), as shown in Supplementary Table 3. None of the innate immune markers showed significant difference in concentrations between MCI and sex across the entire sample. See Fig. 2 and Supplementary Table 3.

### Sex differences in the relationship between CSF innate immune markers and neurodegeneration markers

**Figure 3:**
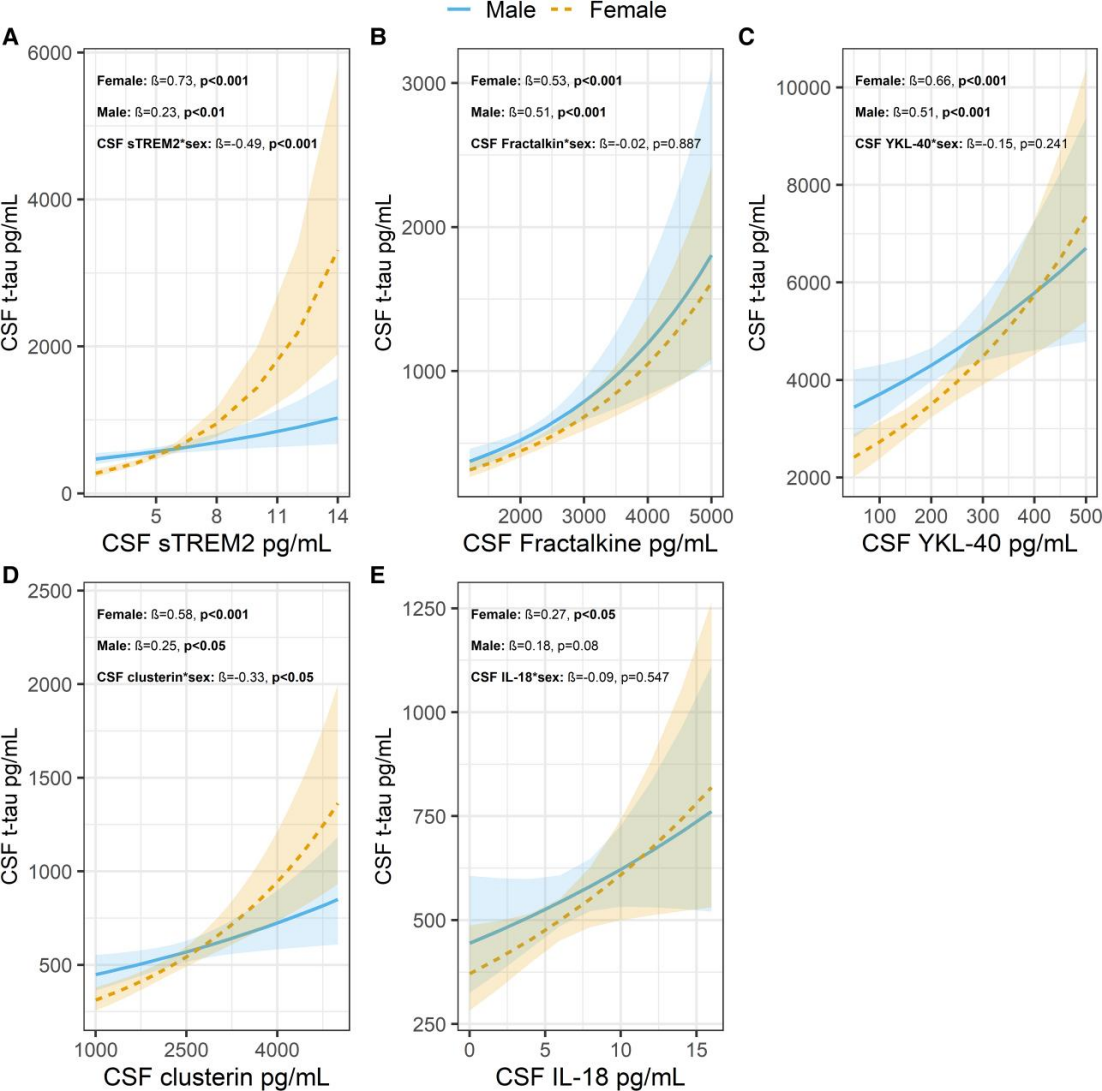
**Sex differences in the relationship between CSF innate immune markers and total tau in amyloid-positive individuals:** Illustrates the differences in the associations between the innate immune markers (**A**) sTREM2, (**B**) fractalkine, (**C**) YKL-40, (**D**) clusterin and (**E**) IL-18 and the neurodegeneration marker total tau (t-tau) in males (blue solid lines) and females (yellow dashed lines) within the amyloid-positive group (A+, *n* = 202). Bands fitted to each regression line show the 95% confidence interval for the estimates. Multiple linear regression analyses were conducted within the A+ cases including an interaction term between sex and innate immune markers, with log-transformed t-tau as the dependent variable. Age and *APOE-ɛ4* were included as covariates. No adjustments for multiple comparisons were made.

Significant associations between CSF t-tau and CSF sTREM2, fractalkine, YKL-40, clusterin, and IL-18 were found, but not for CSF MCP-1, IL-6, IL-10, IFN-γ, or plasma GFAP (see Supplementary Table 4 for details). While higher sTREM2 (Fig. 3A) was associated with higher t-tau in both females (*β*=0.73, *P*  *<* 0.001) and males (*β*=0.23, *P*  *<* 0.01), the association was significantly stronger for females (difference in slopes: *β* = −0.49, *P*  *<* 0.001). A similar sex difference was observed for clusterin (Fig. 3D), where females (*β* = 0.66, *P*  *<* 0.001) demonstrated stronger associations with t-tau than males (*β* = 0.25, *P*  *<* 0.05; difference in slopes: *β* = −0.33, *P*  *<* 0.05). While only females demonstrated a significant association between IL-18 (Fig. 3E) and t-tau (females: *β* = 0.27, *P*  *<* 0.05; males: *β* = 0.18, *P*  *=* 0.08), the difference in slopes was not significant (*β* = −0.09, *P*  *=* 0.547). For fractalkine (Fig. 3B) and YKL-40 (Fig. 3C), the associations were positive and significant for both males and females; however, no sex differences were observed (see Supplementary Table 4).

**Figure 4:**
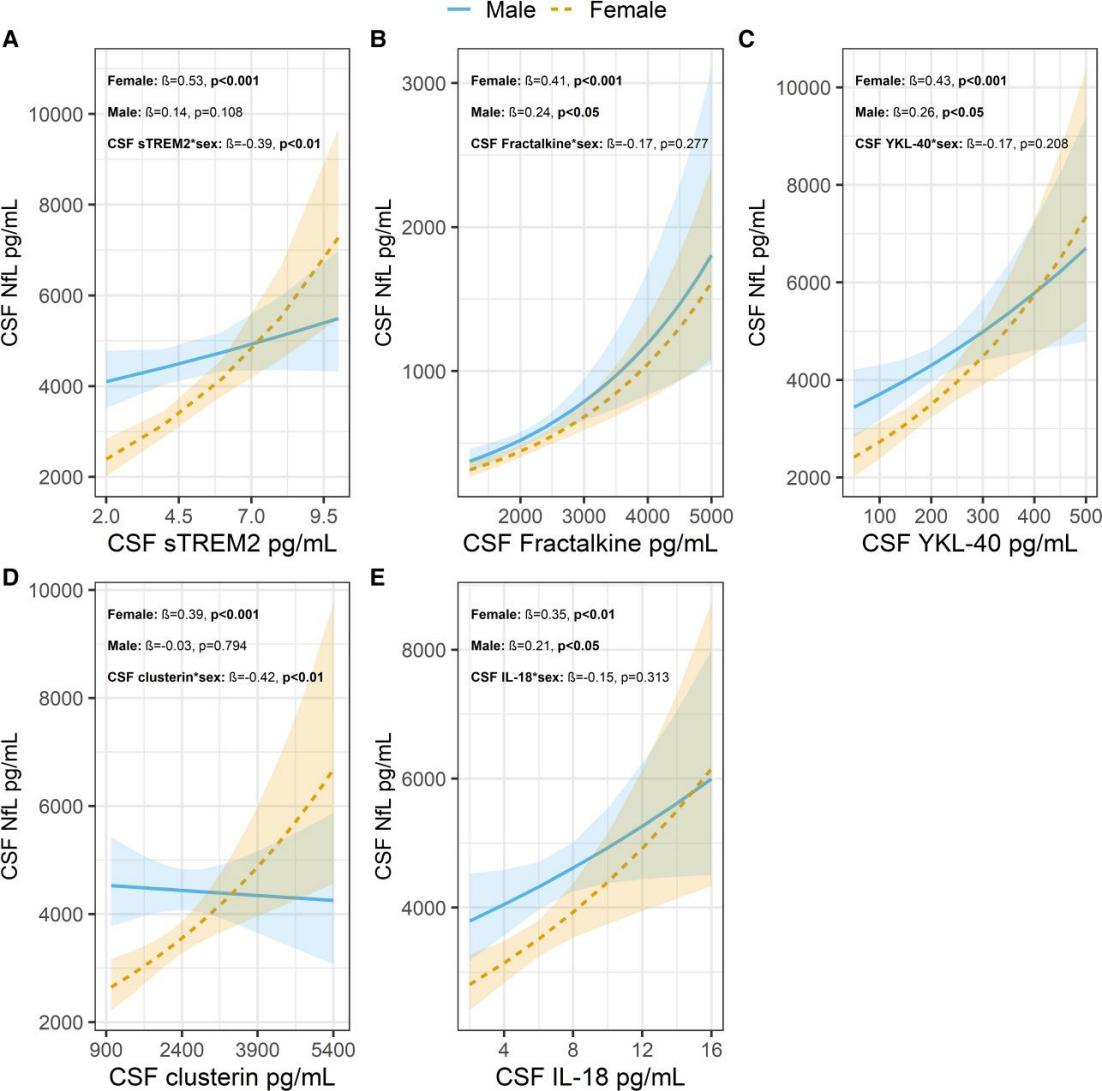
**Sex differences in the relationship between CSF innate immune markers and neurofilament light chain in amyloid-positive individuals:** Illustrates the differences in the associations between the innate immune markers (**A**) sTREM2, (**B**) fractalkine, (**C**) YKL-40, (**D**) clusterin and (**E**) IL-18 and the neurodegeneration marker neurofilament light chain (NfL) in males (blue solid lines) and females (yellow dashed lines) within the amyloid-positive group (A+, *n* = 202). Bands fitted to each regression line show the 95% confidence interval for the estimates. Multiple linear regression analyses were conducted within the A+ cases including an interaction term between sex and the innate immune markers, with log-transformed NfL as the dependent variable. Age and *APOE-ɛ4* were included as covariates. No adjustments for multiple comparisons were made.

For CSF NfL, we found significant positive associations with sTREM2, fractalkine, YKL-40, clusterin, and IL-18, but not for CSF MCP-1, IL-6, IL-10, IFN-γ, or plasma GFAP (see Supplementary Table 5). Males had significantly higher concentrations of CSF NfL in all models (see Supplementary Table 5). However, as with t-tau, we also found that females had stronger associations between sTREM2 (Fig. 4A) and NfL (*β* = 0.53, *P*  *<* 0.001) than males (*β* = 0.14, *P*  *=* 0.108; difference in slope: *β* = −0.39, *P*  *<* 0.01). A similar pattern was observed for clusterin (Fig. 4D), where females (*β* = 0.39, *P*  *<* 0.001) demonstrated stronger associations with NfL than males (*β* = −0.03, *P*  *=* 0.794; difference in slope: *β* = −0.42, *P*  *<* 0.01). For fractalkine (Fig. 4B), YKL-40 (Fig. 4C), and IL-18 (Fig. 4E), we observed numerically stronger associations for females (*β* = 0.41 *P*  *<* 0.001; *β* = 0.43, *P*  *<* 0.001; *β* = 0.35, *P*  *<* 0.01, respectively) than males (*β* = 0.24, *P*  *<* 0.05; *β* = 0.26, *P*  *<* 0.05; *β* = 0.21, *P*  *<* 0.05, respectively) but the differences were not statistically significant (difference in slopes: *β* = −0.17, *P*  *=* 0.277; *β* = −0.17, *P*  *=* 0.208; *β* = −0.15, *P*  *=* 0.313, respectively).

## Discussion

In the present study, we show that females have lower concentrations of the innate immune markers MCP-1, IL-18, and IL-6 in predementia AD, particularly in the MCI stages. While these differences remained significant after FDR adjustments for multiple testing, none of our models showed significant interaction effects between sex; these findings were obtained in *post hoc* analyses. Nevertheless, we observed stronger associations in predementia AD females than in males between the innate immune markers sTREM2 and clusterin and the neurodegeneration markers t-tau and NfL. Together, these findings suggest a potential role for sex-specific immune responses in AD pathology.

While the lower concentrations of MCP-1, IL18 and IL-6 observed in A+ participants should be interpreted with caution, our findings nevertheless align with previous research reporting sex differences in CSF innate immune markers.^24,43^ In a combined cohort of neurological patients without cognitive dysfunction, those with MCI, and those with AD dementia, Brosseron *et al*.^24^ found that males had significantly higher concentrations of several inflammatory CSF proteins, including MCP-1 and IL-6, consistent with our results. Similarly, Ojala *et al*.^43^ reported higher levels of IL-18 in brain tissue from males compared to females in AD, further supporting our CSF findings. Furthermore, in a community sample of older adults (>60 years), Liu *et al*.^44^ found that males had higher levels of blood IL-18 compared to females. Associations between CSF IL-18 and cognition have also been reported, with one study showing that only males exhibited a correlation between higher CSF IL-18 concentrations and CSF t-tau in an MCI group.^43^ Lower CSF innate immune activation in females may have negative implications concerning disease development in females. This is supported by a two-year follow-up study showing that high microglial activation at baseline was associated with slower clinical progression, whereas low microglial activation at baseline was associated with more rapid clinical decline,^4^ along with another study that found an association between reduced microglial activation and rapid cognitive decline in AD.^45^

No sex differences have been observed in Aβ burden,^46,47^ consistent with our findings suggesting that sex differences in innate immune responses may emerge only after the onset of Aβ accumulation and during the prodromal phase of AD.^48^ Both t-tau and NfL were used as markers of neurodegeneration, and our study showed that A+ females had stronger associations with neurodegeneration markers than males, particularly for sTREM2 and clusterin. CSF t-tau is expressed in unmyelinated axons of the cortex and may be more strongly linked to gray matter degeneration.^49,50^ CSF NfL serves as a better marker for neuroaxonal injury,^51,52^ increases with age, and males have higher concentrations of NfL compared to females.^53^ This is supported by our findings, where males exhibit significantly higher concentrations of CSF NfL in all our models. This has previously been attributed to the higher white matter percentage in males compared to females.^11,54^ Previous observations have noted that females, especially those carrying the *APOE-ɛ4* allele, tend to exhibit higher CSF concentrations of both p-tau and t-tau.^46,55,56^ Given the literature and our findings, it is tempting to speculate that some females may experience a relative hypoactivation of the innate immune response during the prodromal phase of AD.

CSF sTREM2 is a microglial activation marker,^57^ and neuroprotective in the context of AD.^58,59^ sTREM2 concentrations are decreased in the presence of Aβ deposition alone, but increased when tau-tangle pathology (p-tau) and neurodegeneration (t-tau) are present.^9,24,57,60-62^ Sex differences in the association between sTREM2 and neurodegeneration have been studied to a limited extent, with mixed results. While two previous studies found no significant association between CSF sTREM2 concentrations and sex,^60,63^ another study reported a significant correlation between CSF sTREM2, t-tau and sex, indicating that males tend to have higher sTREM2 concentrations than females.^24^ This may indicate a potential protective effect in males.

Clusterin is considered neuroprotective in the early stages of AD, helping to promote Aβ clearance and preserve brain volume.^64,65^ Its ability to inhibit plaque formation appears to be dependent on the ratio of clusterin to Aβ.^66^ Decreased CSF clusterin concentrations are found in early stages of AD (Aβ+),^67^ while increased concentrations are seen in later stages associated with tau-tangle pathology and neurodegeneration.^9,67-69^ Furthermore, in a cellular model, clusterin has been suggested to accelerate tau pathology.^70^ Our findings on stronger associations between clusterin and CSF t-tau and NfL in females may suit the fact that females are more disposed to AD. Whether clusterin promotes or delays tau pathology might be dependent on disease stage, and perhaps also sex, but this has not yet been sufficiently investigated.

Sex differences in immune response and microglial activity have also been observed in animal studies.^71-74^ Male rat microglia exhibited stronger immune responses than female rat microglia after being stimulated with an immune challenge.^75^ Murine studies indicate that microglial senescence reduces Aβ clearance, leading to Aβ accumulation.^76,77^ Female mouse brains exhibit larger changes in gene expression^73^ (and the majority were downregulated) at younger ages compared to male mice, resulting in an earlier hypometabolic state.^78^ Since microglia are heterogenous and vary by brain region, age, and species,^73^ changes in microglial function across developmental stages and later life phases, combined with underlying genetic mutations, may influence the progression of AD.

The literature cited above supports our observations of sex differences in innate immune markers and suggests that low innate immune activation may have detrimental effects on AD development. However, the sexual dimorphism in predementia AD reported in this study could also result from direct or indirect effects of sex hormones and genetic differences, with sex hormones potentially influencing gene expression.^79^ Murine studies suggest that oestrogen and testosterone are likely important in the maturation and development of immune cells and immune responses,^80^ and thus cannot be ruled out as contributing factors in humans. However, the inclusion of only postmenopausal women reduces potential biases from cyclic sex hormone variations. Furthermore, differential innate immune gene expression patterns between females and males are expected, as several immune-related genes are located on the X chromosome.^81^ The escape of X chromosome inactivation in females is believed to contribute to the higher prevalence of autoimmune diseases in females.^82^ Additionally, a maternal history of AD has been associated with a greater risk of AD development compared to paternal or no family history.^83-85^

There have been high expectations for anti-amyloid monoclonal antibodies (MABs) as disease modifying treatment of AD, and beneficial effects have been demonstrated for aducanumab,^86,87^ lecanemab^23^ and donanemab.^88^ However, adverse and potentially serious side effects, such as amyloid-related imaging abnormalities (ARIA), have also been reported.^89^  *APOE-ɛ4* carriers have a markedly increased risk of AD, with the risk reported to be higher in females than in males.^46,48,90-92^ While *APOE-ɛ4* carriers are reported to have an increased risk for ARIA-edema (ARIA-E), no significant sex differences have been reported.^89^ However, the generally favourable effect of lecanemab has been reported as less pronounced for females than males (supplementary Van Dyck *et al.*^23^). The promising effects of MABs have been linked to microglial activation and phagocytosis.^93^ It is therefore tempting to speculate whether females may have a reduced effect of MABs due to a diminished immune response in the early stages of AD.

Our study analyzed immune markers in CSF rather than plasma (except for GFAP since it is proven more specific in plasma compared to CSF in context of AD)^94^ in a relatively large cohort (*n* = 285), which may strengthen the validity of our findings. This study is however subject to some limitations. First, while our stringent *post hoc* analyses suggested lower innate immune marker levels in A+ female participants, all but one marker showed non-significant interaction effects in our between-group models. Despite this, we explored these sex differences, as they were partially anticipated based on previous literature. However, these findings should be interpreted with caution, as non-significant interaction effects may reflect either substantial individual variability within sex and Aβ groups or insufficient statistical power to detect a true interaction. Secondly, our study did not include longitudinal data; thus, we cannot ascertain how innate immune markers may change over time between sexes along the AD continuum. Thirdly, we did not assess sex differences in CSF-determined A+ and p-tau negative or A+ and p-tau-positive group. However, we included CSF t-tau and NfL as covariates and assessed linear associations with t-tau and NfL for all markers in our study. Lastly, comorbidities such as autoimmune diseases, cancer, and other conditions could theoretically elevate inflammatory markers and increase the risk of developing AD. In our study, this is accounted for by the inclusion and exclusion criteria, which ruled out several severe diseases. However, participants may have developed relevant conditions after inclusion, though we expect these cases to be rare. We have data on CRP concentrations (normal: *n* = 245, abnormal: *n* = 19, missing: *n* = 21) and leukocyte counts (normal: *n* = 215, abnormal: *n* = 18, missing: *n* = 52). Additionally, participants with autoimmune diseases could potentially influence our results, but their numbers were low (no autoimmune disease: *n* = 264, autoimmune disease: *n* = 9, missing: *n* = 12).

## Conclusion

Our results suggest that the immune system may play a role in differing development and presentation of AD in females versus males, as females may have lower concentrations of CSF innate immune activation markers in participants with established amyloid pathology, and amyloid pathology with MCI, even when adjusting for pertinent CSF markers of neurodegeneration. Moreover, sex differences were also found in CSF immune marker associations to neurodegeneration markers. The observed sex differences in innate immune system markers could contribute to differences in AD risk and development at the predementia stage and may help explain variations in incidence and progression between males and females. Sexual dimorphism may hold the potential for the future development of sex specific treatments for AD.

## Supplementary Material

fcaf161_Supplementary_Data

## Supplementary material

Supplementary Tables 1–5 are available at *Brain Communications* online.
